# Association between post-transplant red cell distribution width and prognosis of kidney transplant recipients

**DOI:** 10.1038/s41598-017-13952-6

**Published:** 2017-10-23

**Authors:** Sehoon Park, Young Hoon Kim, Yong Chul Kim, Mi-Yeon Yu, Jung Pyo Lee, Duck Jong Han, Yon Su Kim, Su-Kil Park

**Affiliations:** 1 Department of Biomedical Sciences, Seoul National University College of Medicine, Seoul, Korea; 2 Department of Surgery, Asan Medical Center, University of Ulsan College of Medicine, Seoul, Korea; 3 Kidney Research Institute, Seoul National University College of Medicine, Seoul, Korea; 4 Department of Internal medicine, Seoul National University Boramae Medical Center, Seoul National University College of Medicine, Seoul, Korea; 5 Department of Internal Medicine, Seoul National University Hospital, Seoul, Korea; 6 Department of Internal Medicine, Asan Medical Center, University of Ulsan College of Medicine, Seoul, Korea

## Abstract

The role of elevated post-transplant red cell distribution width (RDW) as a predictive factor for graft loss remains unclear, although RDW was reported to be significantly associated with poor prognosis in various clinical fields. We performed a retrospective cohort study with 2,939 kidney transplant patients from two tertiary teaching hospitals in Korea. RDW level at transplantation and 3-months post-transplantation were collected. Those with RDW in the upper quartile range were considered to have increased RDW (>14.9%). Death-with-graft-function (DWGF), death-censored graft failure (DCGF), and composite graft loss were assessed as the study outcomes, using multivariable cox proportional hazard model. At the median follow-up duration of 6.6 (3.6–11.4) years, 336 patients experienced graft loss. There were 679 patients with elevated RDW at 3-months post-transplant. Elevated RDW was associated with composite graft loss (adjusted hazard ratio, 1.60, 95% confidence interval, 1.23–2.07, P < 0.001), even after adjusted for hemoglobin and various clinical factors. The 1% increment of post-transplant RDW was also significantly associated with the outcome, regardless of the presence of anemia. The worst prognosis was seen in patients with elevated RDW after transplantation, but not at baseline. Therefore, post-transplant RDW level may be significantly associated with patient prognosis, independent of hemoglobin values.

## Introduction

Red cell distribution width (RDW) is routinely reported in one of the most commonly used panel exams, complete blood cell count (CBC)^1^. RDW is widely used for differential diagnosis of anemia, and detecting early iron deficiency^2,3^. Many recent studies have focused on the association between RDW levels and clinical outcomes, mostly in the field of cardiology^4–14^. The association of elevated RDW and poor prognosis was further confirmed by meta-analyses^15,16^. Still, the mechanism of RDW and its relationship with clinical outcomes has not been fully understood, but associated inflammation, iron deficiency, and/or poor nutritional status may be possible causes^7,17^.

The kidney is an important organ for hematopoiesis. Kidney dysfunction consequently leads to anemia, and other hematologic dysfunction, such as impaired hemostasis^18,19^. Regarding RDW, decreased kidney function is an important clinical factor related to abnormal red cell indices^7^. Moreover, RDW is an important prognosis predictor in those with reduced kidney function^8,9^. Yet, in the kidney transplantation (TPL) field, only a few studies have focused on the predictive value of RDW^12,13^. These studies demonstrated that elevated RDW was related to poor post-TPL outcomes in renal TPL recipients, but the studies had several limitations. In addition, it remains unclear whether post-TPL RDW increment is associated with graft loss.

In this study, we retrospectively analyzed a large cohort of kidney TPL recipients with available RDW levels, and investigated the clinical significance of RDW increment after TPL. Moreover, we collected clinical outcomes, including both death-with-graft-function (DWGF) and death-censored-graft-failure (DCGF), to determine if there was an association between elevated RDW and long-term prognosis.

## Results

### Study population

**Figure 1:**
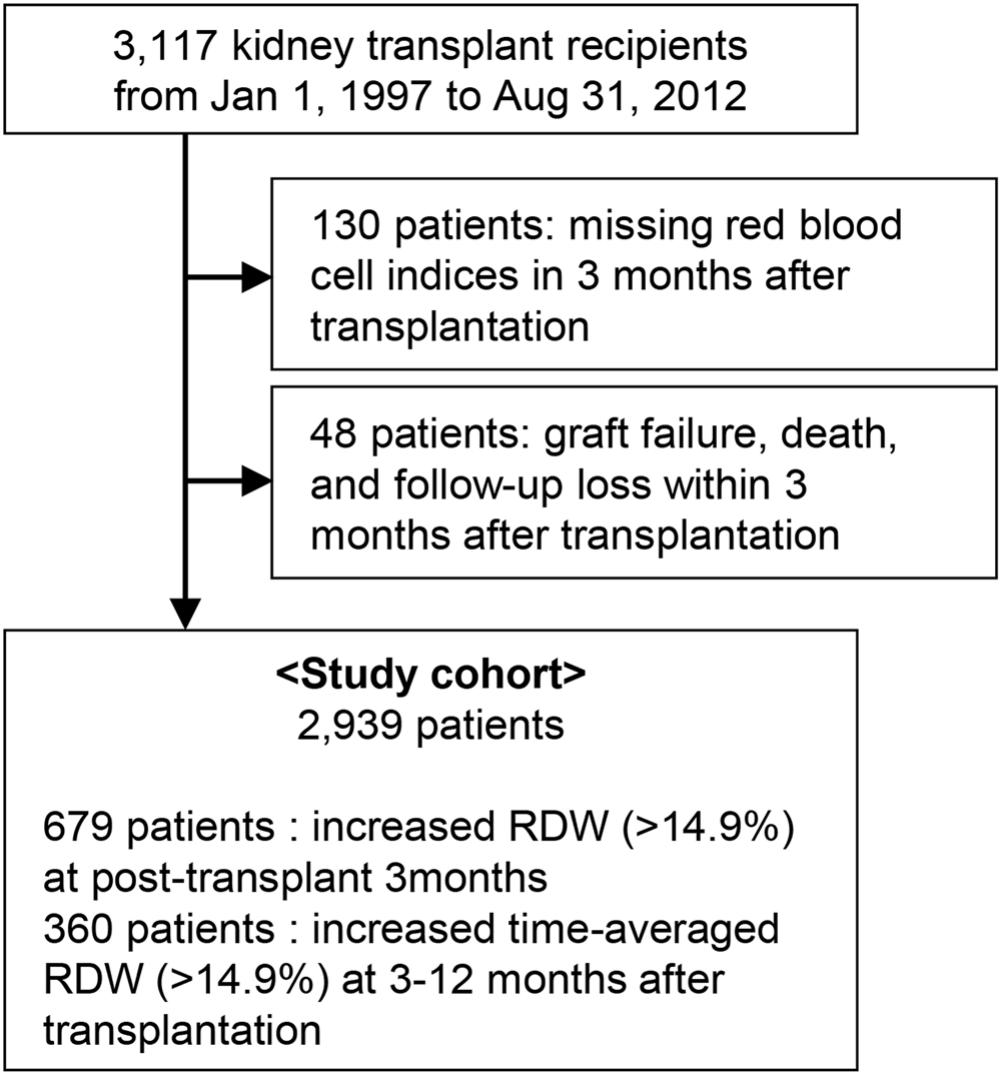
Study population. The flow diagram of the study cohort; RDW, red cell distribution width.

Figure 1 shows the study flow diagram. There were 3,117 patients who received renal TPL that was not part of a multi-organ TPL. After exclusion of those without available RDW levels at 3 months after surgery (N = 130), and those with follow-up or graft loss within 3 months (N = 48), the remaining 2,939 patients were included in the study cohort. Among them, 679 patients had elevated RDW levels (>14.9%) at 3 months post-TPL, and 360 patients had increased post-operative time-averaged RDW values.

### Baseline characteristics

Baseline characteristics according to the presence of elevated RDW levels at 3 months post-operation are shown in Table 1. Patients with high RDW levels were older (P < 0.001), more frequently male (P = 0.003), and had a higher body mass index (BMI) (P = 0.01). End stage renal disease (ESRD) causes also differed between groups. Patients with a RDW > 14.9% more often had diabetes mellitus (P < 0.001) and hypertensive nephropathy (P < 0.001); though, primary glomerulopathy was a relatively uncommon cause of the renal failure in those with high RDW (P < 0.001). History of smoking (P = 0.002) and diabetes mellitus (P = 0.001) were more frequent in patients with elevated RDW; in contrast, the incidence of hypertension was similar between groups (P = 0.28).

**Table 1:** Clinical characteristics according to the presence of increased RDW at post-TPL 3 months.

Characteristics	RDW ≤ 14.9% (n = 2260)	RDW > 14.9% (n = 679)	P value
Recipient characteristics			
Age (years)	41.0 (32.0–50.0)	45 (36.0–53.0)	<0.001
<50	1687 (74.6)	439 (64.7)	
≥50	573 (25.3)	240 (35.3)	
Sex (male)	1306 (57.8)	437 (64.4)	0.003
Body mass index (kg/m^2^)	22.0 (20.1–24.2)	22.4 (20.4–24.6)	0.01
Cause of ESRD			<0.001
Primary glomerulopathy	523 (24.6)	119 (18.6)	
Diabetic nephropathy	264 (12.4)	111 (17.3)	
Hypertensive nephropathy	139 (6.5)	64 (10.0)	
Polycystic kidney disease	82 (3.8)	30 (4.7)	
Unknown or miscellaneous	1123 (52.7)	317 (49.5)	
Smoking history	451 (20.0)	175 (25.8)	0.002
Hypertension	1892 (83.8)	581 (85.6)	0.28
Diabetes mellitus	357 (15.8)	143 (21.1)	0.001
Pre-TPL RDW (%)	13.4 (12.8–14.3)	13.9 (13.1–14.7)	<0.001
Laboratory tests at post-TPL 3 months			
Anemia-related tests			
^a^Hemoglobin (g/dL)	12.5 (11.4–13.5)	11.4 (10.1–12.7)	<0.001
^b^Anemia	1127 (49.9)	502 (73.9)	<0.001
MCV (fL/red cell)	94.1 (90.6–97.8)	97.3 (92.0–103.1)	<0.001
Iron (μg/dL)	75 (44.5–102.5)	62.5 (37.0–100.0)	0.10
Ferritin (μg/L)	254.5 (112.0–426.9)	577.2 (212.1–980.0)	<0.001
TIBC (μg/dL)	243.0 (207.0–274.0)	218.0 (177.0–261.0)	<0.001
TSAT (%)	30.2 (19.8–40.2)	30.1 (17.5–46.3)	0.97
Iron deficiency (TSAT <20%)	28 (25.5)	28 (32.6)	0.35
Serum creatinine (mg/dL)	1.29 (1.00–1.55)	1.28 (1.00–1.60)	0.89
eGFR (mL/min/1.73 m^2^)	57.1 (42.9–84.3)	55.9 (40.0–80.2)	0.02
≥60	1037 (45.9)	293 (43.3)	
30–60	1040 (46.1)	314 (46.4)	
<30	180 (8.0)	69 (10.2)	
Albumin (g/dL)	4.1 (3.8–4.3)	3.8 (3.6–4.1)	<0.001
Hypoalbuminemia (<3.0 g/dL)	17 (0.8)	35 (5.6)	<0.001
C-reactive protein (mg/dL)	0.1 (0.0–0.5)	0.2 (0.1–0.5)	<0.001
Donor characteristics			
Age (years)	39 (30–48)	40 (32–49)	0.06
Sex (male)	950 (42.9)	279 (42.5)	0.87
Relationship			0.007
Living related	1199 (53.7)	327 (48.7)	
Living unrelated	537 (24.0)	156 (23.2)	
Deceased	497 (22.2)	188 (28.0)	
TPL related characteristics			
ABO mismatch	167 (7.5)	46 (7.0)	0.69
Positive cross-match	75 (3.3)	20 (2.9)	0.72
Acute rejection within 3 months	171 (7.6)	75 (11.0)	0.005
Number of HLA mismatch			0.02
Full match	239 (11.1)	55 (8.4)	
Mismatch 1–3	1115 (51.6)	319 (48.7)	
Mismatch 4–6	807 (37.3)	281 (42.9)	
Medication use of			
Tacrolimus	1098 (48.6)	263 (38.7)	<0.001
Cyclosporine	999 (44.2)	314 (46.2)	0.38
Azathioprine	319 (14.1)	114 (16.8)	0.10
Induction therapy	1216 (53.8)	421 (62.0)	<0.001
Treatment for anemia within 3 months			
Use of erythropoietin	89 (3.9)	65 (9.6)	<0.001
Transfusion of RBC	614 (27.2)	266 (39.2)	<0.001
1–2 packs	402 (65.5)	131 (49.2)	<0.001
3–9 packs	189 (30.8)	101 (38.0)	<0.001
≥10 packs	23 (3.7)	34 (12.8)	<0.001

RDW, red cell distribution width, TPL, transplantation, ESRD, end-stage renal disease, MCV, mean corpuscle volume, TIBC, total iron binding capacity, TSAT, transferrin saturation, eGFR, estimated glomerular filtration rate, HLA, human leukocyte antigen. Categorical variables were presented as n (%), and continuous variables were shown as median scores (interquartile ranges). ^a^The hemoglobin values were measured in the same complete blood cell panel exam which reported the RDW values. ^b^Presence of anemia was defined with hemoglobin level <12 g/dL for women and <13 g/dL for men.

Patients with elevated RDW had unfavorable laboratory results compared to patients without elevated RDW, including lower estimated glomerular filtration rate (eGFR) (P = 0.02), hemoglobin (P < 0.001), and albumin (P < 0.001) levels at post-TPL period. C-reactive protein was higher in those with elevated RDW (P < 0.001), but median difference was small. However, although available only in a limited number of patients, TSAT (P = 0.97) or presence of iron deficiency (TSAT < 20%, P = 0.35) was similar between the study groups.

Concerning TPL-related characteristics, the presence of a deceased donor was more common (P = 0.007), more frequent acute rejection was identified (P = 0.005), and a higher human leukocyte antigen (HLA) mismatch level was observed (P = 0.02) in those with elevated RDW values. Lastly, immunosuppressive induction therapy was more commonly performed (P < 0.001), yet, the use of tacrolimus was less frequently identified (P < 0.001) in patients with high RDW. The frequency of use of other treatment regimens, including the use of cyclosporine (P = 0.38) and azathioprine (P = 0.10), were similar between the study groups. In regards of treatment modalities, TPL recipients with elevated RDW levels more commonly received erythropoietin treatment or RBC transfusion (P < 0.001). In addition, the difference in baseline characteristics were also shown; we divided our study patients into tertile RDW subgroups (see Supplementary Table 1).

### Clinical characteristics related to increased RDW levels

In our multivariable analyses (Table 2), older age (adjusted OR 1.01, 95% CI 1.01–1.02, P = 0.002), male sex (adjusted OR 1.71, 95% CI 1.37–2.12, P < 0.001), history of smoking (adjusted OR 1.31, 95% CI 1.03–1.65, P = 0.03), and graft source being from deceased donor (adjusted OR 1.25, 95% CI 1.00–1.55, P = 0.05) were independently associated with the presence of elevated RDW. Patients with 1 g/dL higher hemoglobin level were less likely to have elevated RDW (adjusted OR 0.69, 95% CI 0.65–0.73, P < 0.001). The presence of hypoalbuminemia (adjusted OR 2.18, 95% CI 1.16–4.11, P = 0.02) was associated with the presence of increased RDW. Those who were treated with induction therapy also had high RDW values (adjusted OR 1.49, 95% CI 1.21–1.83, P < 0.001), but tacrolimus was negatively related to increased post-TPL RDW (adjusted OR 0.56, 95% CI 0.46–0.69, P < 0.001).

**Table 2:** Factors related to the increased RDW (>14.9%) at post-TPL 3 months.

Variables	^a^Adjusted OR (95% CI)	P
Age (1-year increment)	1.01 (1.01–1.02)	0.002
Male sex (vs. female)	1.71 (1.37–2.12)	<0.001
Body mass index (1 kg/m^2^ increment)	1.02 (0.99–1.05)	0.24
Smoking (vs. never)	1.31 (1.03–1.65)	0.03
Diabetes mellitus (vs. none)	1.09 (0.85–1.39)	0.49
Post-TPL 3 month eGFR < 30 mL/min/1.73 m^2^ (vs. eGFR ≥ 30 mL/min/1.73 m^2^)	0.75 (0.54–1.05)	0.09
Underlying primary glomerulopathy (.vs other cause of ESRD)	0.83 (0.66–1.06)	0.13
Hemoglobin (1 g/dL increment)	0.69 (0.65–0.73)	<0.001
Hypoalbuminemia (<3.0 g/dL)	2.18 (1.16–4.11)	0.02
Deceased donor (.vs other donor relationship)	1.25 (1.00–1.55)	0.05
Number of mismatched HLA (one antigen increment)	1.02 (0.96–1.08)	0.57
Acute rejection within 3 months (vs. none)	1.17 (0.86–1.61)	0.32
Induction therapy (vs. none)	1.49 (1.21–1.83)	<0.001
Use of tacrolimus (vs. no use)	0.56 (0.46–0.69)	<0.001

OR, odds ratio, CI, confidence interval, eGFR, estimated glomerular filtration rate, ESRD, end stage renal disease, HLA, human leukocyte antigen, TPL, transplantation. Missing data was imputed by multiple imputation by classification and regression trees (CART) method. ^a^All variables in the Table 2 were simultaneously adjusted, and odds ratio/confidence interval of each characteristic was shown.

### Association between post-transplant RDW and graft loss

During the 6.6 (3.6–11.4) year median follow-up period, there were 95 cases of DWGF and 241 cases of DCGF. Among them, 66 cases of DWGF and 223 cases of DCGF occurred more than a year after the TPL.

When we assessed the composite graft loss, in addition to several known predictive factors, elevated 3-month post-TPL 3 RDW (>14.9%) was significantly associated with increased risk for graft loss (hazard ratio [HR] 2.03, 95% CI 1.60–2.58, P < 0.001) (Table 3). Per the multivariable analyses, DM (adjusted HR 1.74, 95% CI 1.28–2.38, P < 0.001), graft from deceased donor (adjusted HR 1.56, 95% CI 1.20–2.03, P < 0.001), and induction therapy (adjusted HR 0.72, 95% CI 0.54–0.96, P = 0.02) were all characteristics associated with composite graft loss in the study cohort. Increased RDW remained as an independent risk factor for composite graft loss (adjusted HR 1.60, 95% CI 1.23–2.07, P < 0.001). Moreover, the association with post-TPL RDW, both the increment over the certain value (14.9%) and 1% higher level, with graft loss was evident regardless of the presence of co-existing anemia (see Supplementary Table 2) or the use of erythropoietin treatment or RBC transfusions (see Supplementary Table 3

**Table 3:** Variables associated with composite graft loss in the study cohort.

Variables	Univariable analyses		Multivariable analyses	
	HR (95% CI)	P	^a^Adjusted HR (95% CI)	P
Increased post-TPL RDW (>14.9%)	2.03 (1.60–2.58)	<0.001	1.60 (1.23–2.07)	<0.001
Age (1-year increment)	1.02 (1.01–1.03)	<0.001	1.00 (0.99–1.01)	0.76
Male sex (vs. female)	1.37 (1.08–1.74)	0.01	1.26 (0.95–1.66)	0.11
Body mass index (1 kg/m^2^ increment)	1.06 (1.02–1.09)	0.003	1.24 (0.98–1.07)	0.26
Smoking (vs. never)	1.29 (0.98–1.69)	0.07	1.14 (0.85–1.54)	0.38
Diabetes mellitus (vs. none)	1.94 (1.46–2.57)	<0.001	1.74 (1.28–2.38)	<0.001
Post-TPL 3 month eGFR < 30 mL/min/1.73 m^2^ (vs. eGFR ≥ 30 mL/min/1.73 m^2^)	1.78 (1.22–2.61)	0.003	1.37 (0.91–2.08)	0.13
Underlying primary glomerulopathy (.vs other cause of ESRD)	0.69 (0.50–0.95)	0.02	0.83 (0.60–1.16)	0.28
Hemoglobin (1 g/dL increment)	0.89 (0.83–0.95)	<0.001	0.93 (0.87–1.00)	0.06
Hypoalbuminemia (<3.0 g/dL)	2.12 (1.13–3.98)	0.02	1.36 (0.71–2.59)	0.35
Deceased donor (.vs other donor relationship)	1.63 (1.27–2.08)	<0.001	1.56 (1.20–2.03)	<0.001
Number of mismatched HLA (one antigen increment)	1.00 (0.79–1.27)	0.99	1.07 (0.99–1.16)	0.09
Acute rejection within 3 months (vs. none)	1.50 (1.05–2.16)	0.03	1.20 (0.83–1.75)	0.34
Induction therapy (vs. none)	0.92 (0.71–1.19)	0.51	0.72 (0.54–0.96)	0.02
Use of tacrolimus (vs. no use)	1.00 (0.79–1.27)	0.99	1.03 (0.80–1.33)	0.82

HR, hazard ratio, CI, confidence interval, DM, diabetes mellitus, eGFR, estimated glomerular filtration rate, ESRD, end stage renal disease, HLA, human leukocyte antigen, RDW, red cell distribution width, TPL, transplantation. Missing data was imputed by multiple imputation by classification and regression trees (CART) method, and hazard ratio and associated confidence of each characteristic was shown. ^a^The multivariable cox regression model was adjusted for all variables in the Table 3, which were the baseline characteristics which were significantly different according to presence of increment RDW.

).

### Death-with-graft-function and death-censored-graft-failure

**Figure 2:**
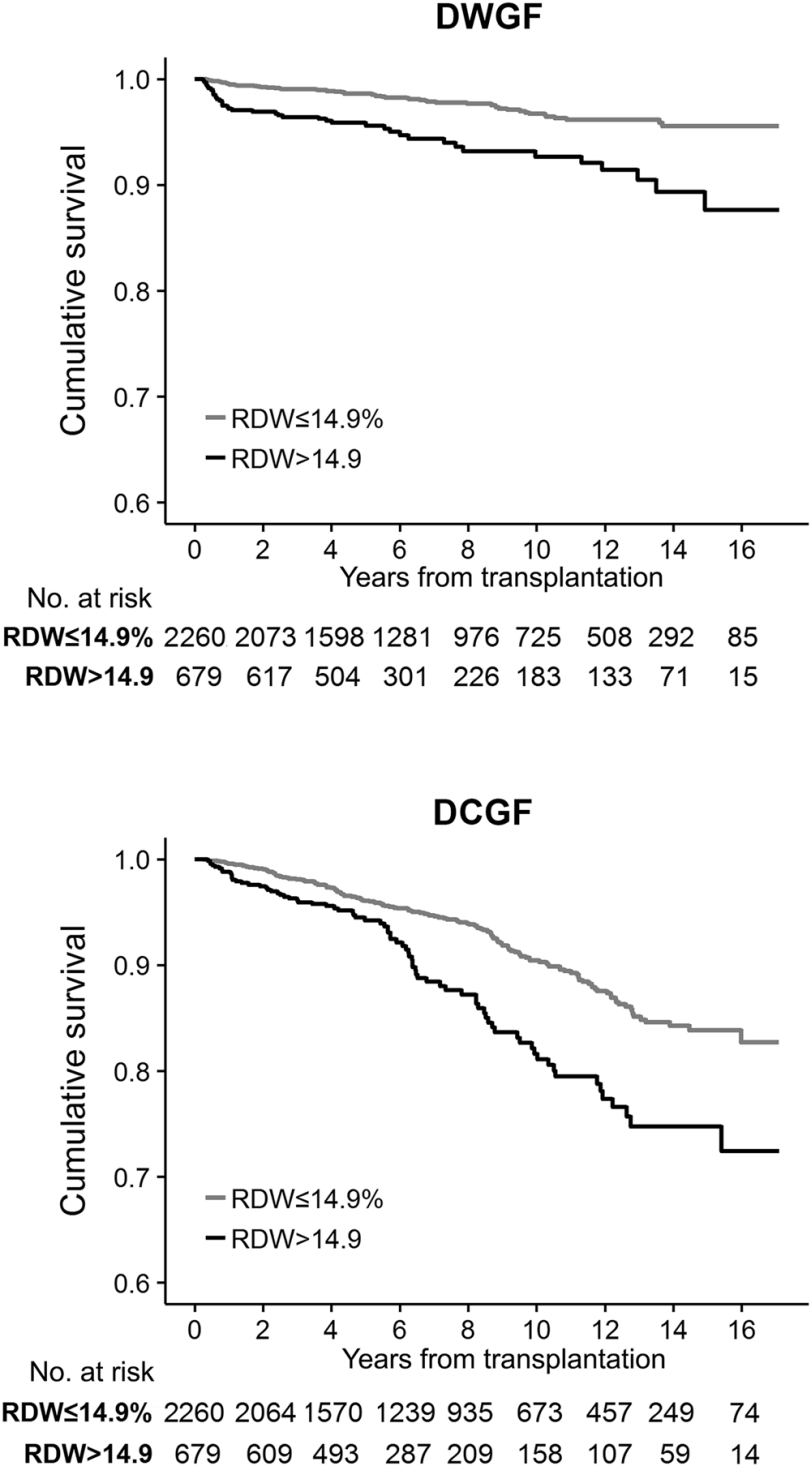
DWGF and DCGF, according to the presence of increased post-transplantation RDW. Cumulative survival curve of the study population, y-axis indicated the cumulative survival and x-axis indicated the years from transplantation. The upper graph shows the DWGF, and the lower graph shows the DCGF. The black line indicates the cumulative survival of increased RDW (>14.9%) level at post-transplant 3 month, the grey line indicates the cumulative survival of others with non-elevated RDW (≤14.9%). The tables presenting the number of patients at risk were shown below the survival curves; RDW, red cell distribution width; DWGF, death-with-graft-function, DCGF, death-censored-graft-failure.

**Figure 3:**
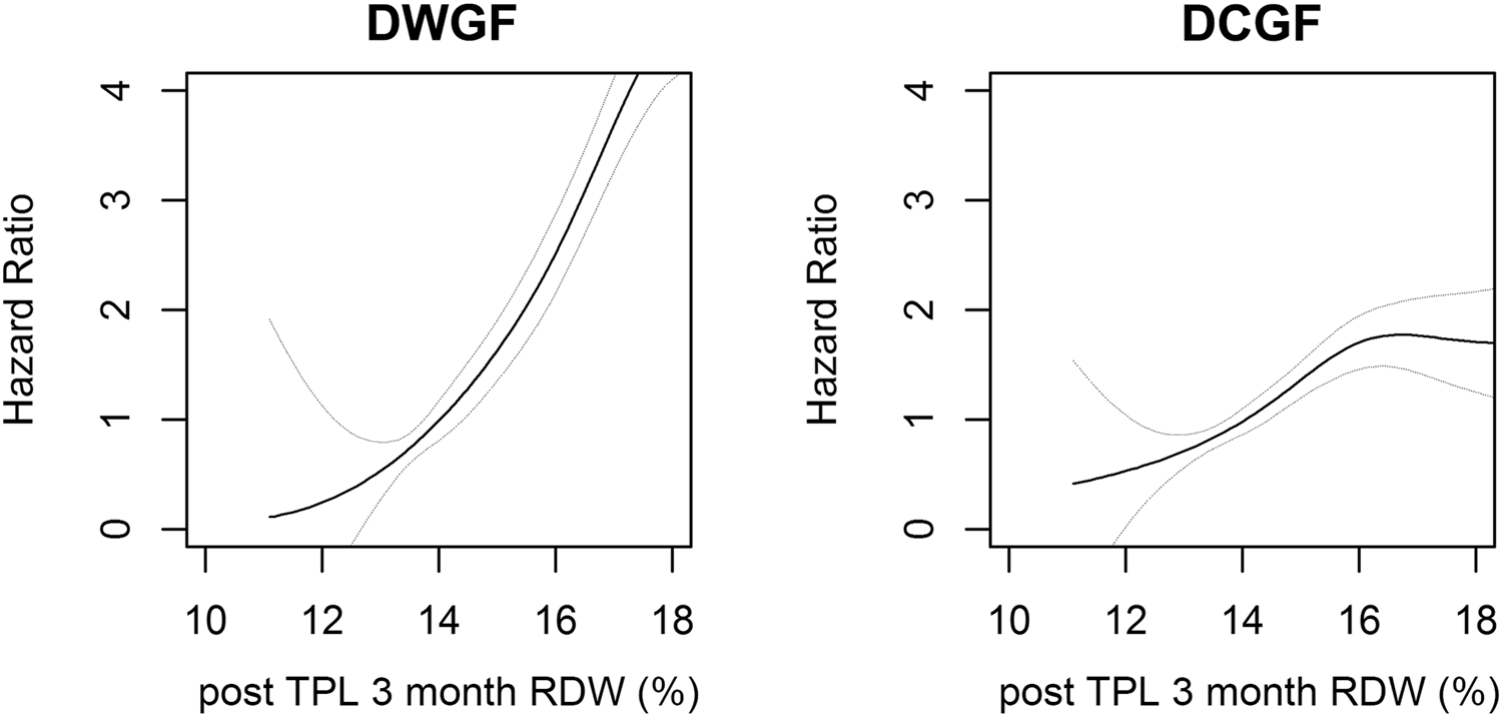
The association between post-TPL RDW values and clinical outcomes. The panelized smoothing splines showing the relationship between RDW levels at post-TPL 3 months and hazard ratios. Left graph indicated the graph using risk of DWGF as binomial outcome and right graph indicated the graph using DCGF as the outcome. The linear line is the associated hazard ratios and upper and lower grey line indicated the 95% confidence intervals; DWGF, death-with-graft-function, DCGF, death-censored-graft-failure; TPL, transplantation; RDW, red cell distribution width.

Next, we analyzed the two outcomes, DWGF and DCGF, separately. The patients who had post-TPL RDW increment showed prominently worse DWGF and DCGF (Fig. 2), and this association also remained to be significant when we divided the study population according to tertile RDW ranges (see Supplementary Fig. 1). Both elevated RDW (>14.9%) at 3-months post-TPL and time-averaged RDW for a certain period were significantly associated with DWGF and DCGF, as well as with 1% increment RDW levels (Table 4). In contrast, when adjusted together, higher hemoglobin level was not significantly related to post-TPL prognosis, except for the association of time-averaged hemoglobin value with DCGF (see Supplementary Table 4). Overall, higher RDW level was associated with increased risk of both DWGF and DCGF (Fig. 3).

**Table 4:** The association between RDW and hemoglobin levels and DWGF/DCGF.

	Univariable analyses		Complete case analyses			^a^Models with missing imputation	
	HR (95% CI)	P	^b^Adjusted HR (95% CI)	P		^b^Adjusted HR (95% CI)	P
DWGF							
Increased RDW (>14.9%)							
Values at 3 months after TPL	2.77 (1.84–4.16)	<0.001	1.88 (1.16–3.05)	0.01		1.75 (1.12–2.73)	0.01
^c^Time-averaged, 3–12 months	1.88 (1.00–3.51)	0.05	1.63 (0.84–3.17)	0.15		1.53 (0.80–2.93)	0.20
RDW increment 1%							
Values at 3 months after TPL	1.27 (1.17–1.38)	<0.001	1.19 (1.07–1.33)	0.002		1.18 (1.06–1.31)	0.002
^c^Time-averaged, 3–12 months	1.40 (1.19–1.66)	<0.001	1.38 (1.13–1.69)	0.001		1.32 (1.08–1.62)	0.006
DCGF							
Increased RDW (>14.9%)							
Values at 3 months after TPL	1.85 (1.41–2.42)	<0.001	1.66 (1.20–2.30)	0.002		1.62 (1.21–2.18)	0.001
^c^Time-averaged, 3–12 months	1.73 (1.22–2.47)	0.002	1.59 (1.08–2.34)	0.02		1.56 (1.08–2.26)	0.02
RDW increment 1%							
Values at 3 months after TPL	1.18 (1.11–1.26)	<0.001	1.17 (1.08–1.27)	<0.001	1.18 (1.09–1.27)		<0.001
^c^Time-averaged, 3–12 months	1.29 (1.17–1.43)	<0.001	1.23 (1.10–1.37)	<0.001	1.23 (1.10–1.37)		<0.001

HR, hazard ratio, CI, confidence interval, RDW, red cell distribution width, TPL, transplantation, DWGF, death-with-graft-function, DCGF, death-censored graft failure. ^a^Multiple imputation by CART (classification and regression trees) was performed. ^b^Adjusted for age (continuous, years), sex, smoking history, eGFR (categorical, <30, 30–60, ≥60), post-transplant RDW (continuous, %), hypoalbuminemia (categorical, serum albumin <3.0 g/dL), presence of acute rejection (within post-TPL 3 month), baseline diabetes mellitus, hypertension, whether induction therapy was performed, medication use of tacrolimus, donor relationship (categorical, deceased or living). ^c^Calculated time-averaged values (Hemoglobin, eGFR, albumin and RDW) between 3–12 months from operation were used in the analyses. In the analyses using the time-averaged values, the mortality or graft failure cases before 12 months were not included.

### RDW changes from before surgery to post-TPL period

When considering baseline values, those who had elevated RDW only after the kidney TPL, but not at baseline, showed significantly worse outcomes when compared with those without pre- or post-TPL red cell indices abnormality, both in terms of DWGF (adjusted HR 1.77, 95% CI 1.10–2.83, P = 0.02) and DCGF (adjusted HR 1.65, 95% CI 1.21–2.26, P = 0.002) (Table 5). We found that a 1% increment of post-TPL RDW rise from baseline was significantly associated with clinical outcomes, with the relationship remaining valid in both analyses, when adjusted for the absolute hemoglobin value at 3-month post-TPL or amount of hemoglobin change (see Supplementary Table 5

**Table 5:** Analysis with subgroups divided by presence of increment in pre- or post-transplantation RDW.

	Univariable analyses		Complete case analyses		^a^Models with missing imputation	
	HR (95% CI)	P	^b^Adjusted HR (95% CI)	P	^b^Adjusted HR (95% CI)	P
DWGF						
Pre/post-TPL RDW ≤ 14.9% (N = 1941)	Reference	—	Reference	—	Reference	—
only pre-TPL RDW > 14.9% (N = 319)	0.71 (0.28–1.79)	0.47	0.59 (0.21–1.64)	0.31	0.67 (0.26–1.68)	0.39
only post-TPL RDW > 14.9% (N = 547)	2.77 (1.80–4.28)	<0.001	1.89 (1.13–3.16)	0.02	1.77 (1.10–2.83)	0.02
Pre/post-TPL RDW > 14.9% (N = 132)	2.17 (0.93–5.08)	0.07	1.36 (0.57–3.29)	0.49	1.26 (0.53–3.02)	0.60
DCGF						
Pre/post-TPL RDW ≤ 14.9% (N = 1941)	Reference	—	Reference	—	Reference	—
only pre-TPL RDW > 14.9% (N = 319)	0.85 (0.52–1.41)	0.54	0.83 (0.48–1.45)	0.51	0.90 (0.54–1.50)	0.69
only post-TPL RDW > 14.9% (N = 547)	1.89 (1.42–2.53)	<0.001	1.70 (1.20–2.41)	0.003	1.65 (1.21–2.26)	0.002
Pre/post-TPL RDW > 14.9% (N = 132)	1.46 (0.79–2.69)	0.23	1.33 (0.69–2.58)	0.39	1.37 (0.73–2.56)	0.33

HR, hazard ratio, CI, confidence interval, TPL, transplantation, RDW, red cell distribution width, DWGF, death-with-graft-function, DCGF, death-censored graft failure. Those without available pre-TPL RDW levels (59 cases) were considered not to have increased RDW values before transplantation. ^a^Multiple imputation by CART (classification and regression trees) was performed. ^b^Adjusted for age (continuous, years), sex, smoking history, eGFR (categorical, <30, 30-60, ≥60), post-transplant RDW (continuous, %), hypoalbuminemia (categorical, serum albumin <3.0 g/dL), presence of acute rejection (within post-TPL 3 month), baseline diabetes mellitus, hypertension, whether induction therapy was performed, medication use of tacrolimus, donor relationship (deceased)

).

### Other anemia-related tests and post-TPL complications

To further examine the possible underlying mechanism for the above findings, we assessed whether significant differences existed in iron, ferritin, total iron binding capacity (TIBC), or transferrin saturation (TSAT) values, despite their limited availability, according to the RDW levels. Although iron levels, TSAT, or iron deficiency (TSAT < 20%) were similar between the subgroups, ferritin was significantly higher (P < 0.001) and, in contrast, TIBC was lower (P < 0.001), in those with elevated RDW (Table 1). In addition, patients with increased RDW only at the post-TPL period but not at baseline, who had worst prognosis among the subgroups, also showed similar results, as they had significantly higher ferritin (P < 0.001) and lower TIBC (P < 0.001) levels than did other subgroups (see Supplementary Table 5).

In addition, patients who had RDW increment in post-TPL period but not at baseline had higher incidence of post-TPL infectious complications (see Supplementary Table 6). Numbers of patients with reported malignancy after TPL was not significantly different among the subgroups. Next, we added the variable determined by the presence of at least one major infection (pneumonia, urinary tract infection, BK virus or parvovirus, and cytomegalovirus) in our multivariable model, accepting the limitations of such definition. In the model, the association between elevated RDW only at the post-TPL period and composite graft loss remained significant (adjusted HR 1.51, 95% CI 1.14–2.01, P = 0.004).

## Discussion

We identified that post-TPL RDW, a routinely-reported laboratory value in the CBC profile, was significantly associated with graft loss of kidney TPL recipients. The increment of RDW after TPL was an important predictor for patient prognosis. Our study is the first study to demonstrate the predictive value of post-TPL RDW and RDW increment on the two most important outcomes of kidney TPL recipients, DWGF and DCGF, in a large TPL cohort with a long-term follow-up duration.

The RDW has primarily been used for differential diagnosis of anemia or iron deficiency^2,3^, but after the anemia assessment was done, the RDW value was more likely to be overlooked. However, in recent studies, the predictive value of RDW in clinical practice has widely been examined in the field of cardiology^4–7,14,20^, and meta-analyses have confirmed its significant association with patient prognosis^15,16^. Few studies regarded that the clinical significance of elevated RDW levels was not only limited to those with cardiovascular diseases^8,12,13,21–26^, and some studies even demonstrated that RDW was related to the prognosis of people in the general community^10,11,26^. In the era of kidney TPL, a few studies regarded RDW as a risk factor for poor prognosis, but they were limited due to non-standardized date of collection of RDW levels, and the lack of DCGF assessment, one of the most important “hard outcomes” for TPL recipients^12,13^. In the current study, we identified a significant association between elevated RDW and patient prognosis, both including DWGF and DCGF, with the most long-term follow-up duration among studies regarding RDW and post-TPL prognosis. Moreover, its predictive value was deemed significant independently from hemoglobin levels and other several important predictive factors, regardless of the presence of established anemia or the history of erythropoietin treatment or RBC transfusion. The worst prognosis was shown in those with a relatively normal baseline RDW, but elevated RDW post-TPL, indicating that RDW change could be a prognostic biomarker for TPL recipients.

Several mechanisms could be proposed for the poor prognosis in those with elevated RDW. First, RDW and its relationship with inflammation should be considered^7,15,16^. In our study population, patients with increased RDW also had higher serum ferritin levels, an acute-phase reactant, implying that inflammation might have some role on poor post-TPL prognosis of those with high RDW. In otherwise, other iron-deficiency related factors were not prominently different according to the presence of RDW increment. Also, patients with RDW increment had a higher incidence of post-TPL infection complications. Therefore, elevated acute phase reactant and more common inflammatory episodes suggest that inflammation may be one of the key mechanisms of worse prognosis in patients with elevated RDW. Namely, higher RDW may be a marker of the ongoing inflammatory process in TPL recipients. Second, decreased TIBC and high ferritin levels indicate that iron mobilization was impaired in those patients, and, indeed, a low hemoglobin level was significantly correlated with increased RDW. Considering that anemia has been known to be an important risk factor for poor TPL prognosis, RDW could be an indicator for poor hematopoiesis profile, even from the early stages of anemia^3,27,28^. Third, increased RDW and its association with low serum albumin or ferritin levels implies that RDW increment was associated with poor nutritional status^7^. Lastly, elevated RDW itself could have indicated a worse graft status, as the kidney itself is an important organ for hematopoiesis^7,29^. Overall, we believe that RDW could be an important laboratory value, as the level could reflect multiple factors, as mentioned above, related to renal TPL recipients’ prognosis.

In our study, there were several characteristics related to elevated RDW levels, with most of these variables associated characteristics also shown in previous studies, including older age, male sex, impaired renal function, decreased hemoglobin levels, and presence of hypoalbuminemia^10,12,13,30^. A history of smoking was reported to be related to increased RDW in the general population^10,11^. Among the TPL-specific characteristics, the presence of a deceased donor was significantly associated with the presence of increased RDW levels, and this factor was first reported in the current study^12,13^. The impact of immunosuppressive agents on RDW levels could hardly be confirmed because the treatment regimen could not have been standardized, although the use of tacrolimus and induction immunotherapy was significantly associated with RDW levels. Comparison of the effects of several immunosuppressive agents on red blood cell profiles warrants further study.

There were several limitations in the current study. First, confounding bias could be possible due to the study’s retrospective nature, as unincluded factors which could affect the RDW levels and post-TPL prognosis might be present. However, considering the results of studies from various clinical fields^15,16^, and its significant association with poor prognosis in the various analyses we performed, RDW should be considered in relation to the patient prognosis. Second, only a small number of patients had available parameters indicating iron storage status and C-reactive protein levels; therefore, the results of the subset do not represent the entire study sample. Third, the underlying mechanism of the poor prognosis in those with elevated RDW could not be confirmed by our study; therefore, we could not suggest possible management strategies for those with high RDW. Inflammatory reaction may be one of the major mechanisms of reported association, but, as we could not quantify episodes of infection or rejection, this should be confirmed in additional study. Even a RDW value may be merely a marker of severe inflammation or other poor underlying condition, it could be a commonly measured laboratory value that is worth recognizing. Lastly, the cut-off value for increased RDW is not well established in TPL recipients, and the upper quartile value was slightly higher than the cohorts investigated in other studies. However, as many of the clinical factors related to elevated RDW are relatively common in TPL recipients compared to the general population, we accepted the small difference in the upper quartile RDW value. Also, considering that the analysis with RDW increment of 1% or tertile ranges showed similar results, the clinical significance of RDW might truly exist.

In conclusion, post-TPL RDW was significantly associated with the prognosis of kidney TPL recipients. Clinicians should not overlook RDWs predictive value, and, especially, should consider prompt assessment to prevent possible poor prognosis in those with RDW increments after TPL.

## Methods

### Ethical considerations

This study was conducted in accordance with the principles of the Declaration of Helsinki. The clinical and research activities being reported are consistent with the Principles of the Declaration of Istanbul as outlined in the ‘Declaration of Istanbul on Organ Trafficking and Transplant Tourism’. The study design was approved by the institutional review board (IRB) of Asan Medical Center (S2017–0355–0001) and Seoul National University Hospital (H-1702–018–829). Under IRB approval, the informed consent was waived.

### Study design and study population

The study was a bicenter, retrospective cohort study that included adult (≥18 years old) patients who received their first kidney TPL from January 1, 1997 to August 31, 2012 at two tertiary teaching hospitals in Korea. Patients without available red blood cell indices after 3 months from the surgery, who experienced follow-up loss, and graft loss within 3 months after the TPL were excluded. We chose >14.9% as the criterion for elevated RDW, as it was the upper quartile RDW at 3-months post-TPL in our study cohort, and was similar with cut-off values in previous studies^14,16,20,26^. As the definition of elevated RDW is not well-established, an association of a 1% increment in RDW level with clinical prognosis was additionally investigated. The clinical significance of time-averaged RDW level from 3 to 12 months post-TPL was also evaluated.

### Data collection

The following demographic and clinical characteristics were collected: age, sex, and BMI. Smoking history, presence of hypertension and diabetes mellitus, and the causes of ESRD, were recorded. TPL-related information, including donor age, sex, donor relationship with the recipients (i.e., living-related, living-unrelated, deceased), presence of ABO mismatch, flow cytometric cross-match results, number of HLA mismatch, and the presence of biopsy-confirmed acute rejection within the first 3 months were collected. Information on immunosuppressive regimen including induction treatment (e.g., basiliximab or antithymocyte globulin), tacrolimus, cyclosporine, and azathioprine was included. The information of usage of erythropoietin therapy or red blood cell transfusions were included. Numbers of patients with documented infection events of the following categories were recorded: urinary tract infection, pneumonia, BK virus or parvovirus infection, and cytomegalovirus infection. Also, reported malignancy after transplantation was collected.

We retrieved the laboratory results from the time of TPL, and those nearest the post-TPL periods of 3, 6, 9, and 12 months. The laboratory results of serum creatinine (sCr), calculated eGFR by the MDRD equation^31^, hemoglobin, RDW, median corpuscular volume, albumin, C-reactive protein, iron, ferritin, TIBC, and TSAT were obtained at each time point. Hemoglobin values and RDW levels were reported from the same CBC panel exam, and measured by Sysmex XE-2100 hematology analyzer. Lab results suspected to be affected by technical hemolysis were excluded.

Information of two major treatment modalities for anemia, use of erythropoietin and RBC transfusion, was collected. The amount of RBC transfusion was recorded in units by number of packs transfused.

In the final dataset, the following information was missing: BMI (123 cases), ESRD cause (167 cases), hypertension (one case), ABO mismatch (58 cases), donor relationship (35 cases), level of HLA mismatch (123 cases), pre-TPL RDW and hemoglobin (59 cases), 3-month post-TPL albumin (250 cases), C-reactive protein (1696 cases), and eGFR (six cases). Laboratory results for iron, ferritin, TIBC, and TSAT values at 3 months after TPL were available in 197, 207, 470, and 196 cases, respectively, and when the duration was extended to one year the values were available in 347, 363, 601, and 345 cases, respectively.

### Outcome measures

DWGF and DCGF were collected, as these outcomes were considered “hard outcomes” of kidney TPL recipients. The composition of DWGF and DCGF was defined as a composite graft loss, and primarily assessed. Each DWGF and DCGF was additionally analyzed individually. The follow-up loss events were censored, and the median follow-up duration of the study cohort was identified as 6.6 (3.6–11.4) years.

### Statistical analyses

Categorical variables were summarized using frequencies (percentages) and were analyzed via chi-square tests. Continuous variables were summarized as median scores (interquartile ranges) and were analyzed by Mann-Whitney U test, as all continuous variables that were not normally distributed according to Shapiro-Wilk test.

Multivariable logistic regression analysis was used to evaluate the characteristics related to elevated RDW levels. Additionally, the Cox proportional hazard model was used to investigate whether patient prognosis was independently related to RDW levels. Multivariable models for assessment of composite graft loss were adjusted for characteristics that were significantly different at baseline. The graft loss was also assessed in each subgroup, divided by the presence of anemia (hemoglobin <12 g/dL for women and <13 g/dL for men) at 3 months post-TPL or the usage of anemia treatment, including erythropoietin therapy and RBC transfusions.

DWGF and DCGF outcomes were analyzed using the Kaplan-Meier survival curve with log-rank test and plotted according to RDW ranges. The relationships between RDW values and risk of each outcome were additionally plotted using the penalized smoothing spline method. The multivariable cox proportional hazard model for each outcome was adjusted with variables that were independently related to increased post-TPL RDW levels or were considered clinically relevant. The analyses for both DWGF and DCGF were performed separately using the RDW levels at 3 months from TPL and the time-averaged values from post-TPL 3 months to 12 months, considering the RDW value as a time-dependent variable. For the analyses using time-averaged RDW levels, time-averaged hemoglobin, eGFR, and albumin values were adjusted for the multivariable model, in addition to other variables, and those with follow-up loss or graft loss within 12 months after TPL were not considered. Next, whether the RDW rise from baseline to 3 months after TPL was associated with each outcome was assessed adjusting for hemoglobin change during the same period.

We performed complete case analyses, as well as analyses with missing data imputation by the classification and regression trees (CART) method using the R ‘mice’ package, combining results from five imputed datasets using the ‘pool’ function. All statistical analyses were performed using the R package (version 3.2.6, the R foundation). Two-sided p values less than 0.05 indicated statistical significance.

### Data availability statement

The datasets generated during and/or analyzed during the current study are available from the corresponding author on reasonable request.

## Electronic supplementary material

Supplementary materials
