# Supplementary material for: Association between post-transplant red cell distribution width and prognosis of kidney transplant recipients
Source: Sci Rep. 2017 Oct 23;7:13755. doi: 10.1038/s41598-017-13952-6 (PMC5653739; doi:10.1038/s41598-017-13952-6)
Supplement: Supplementary file 1 — Supplementary materials [file 41598_2017_13952_MOESM1_ESM.pdf]

Association between post-transplant red cell distribution width and prognosis of kidney transplant recipients

Sehoon Park[1], Young Hoon Kim[2], Yong Chul Kim[3], Mi-Yeon Yu[3], Jung Pyo Lee[4], Duck Jong Han[2], Yon Su Kim[1,5], and Su-Kil Park[6]

Author affiliations

[1]Department of Biomedical Sciences, Seoul National University College of Medicine, Seoul, Korea

[2]Department of Surgery, Asan Medical Center, University of Ulsan College of Medicine, Seoul, Korea

[3]Kidney Research Institute, Seoul National University College of Medicine, Seoul, Korea

[4]Department of Internal medicine, Seoul National University Boramae Medical Center, Seoul National University College of Medicine, Seoul, Korea

[5]Department of Internal Medicine, Seoul National University Hospital, Seoul, Korea

[6]Department of Internal Medicine, Asan Medical Center, University of Ulsan College of Medicine, Seoul, Korea

Supplementary Table 1. Clinical characteristics according to the tertile RDW ranges.

	RDW < 13.4% (n=983)	14.6% > RDW ≥ 13.4% (n=1030)	RDW ≥ 14.6% (n=926)	P value
Recipient characteristics				
Age (years)	39 (30-47)	43 (34-51)	45 (36-53)	< 0.001
< 50	803 (81.7)	724 (70.3)	599 (64.7)	
≥ 50	180 (18.3)	306 (29.7)	327 (35.3)	
Sex (male)	544 (55.3)	58.6 (60.4)	595 (64.3)	< 0.001
Body mass index (kg/m^2)	21.5 (19.8-23.9)	22.3 (20.4-24.4)	22.5 (20.4-24.7)	< 0.001
Cause of ESRD				< 0.001
Primary glomerulopathy	258 (27.8)	228 (23.5)	156 (17.9)	
Diabetic nephropathy	93 (10.0)	131 (13.5)	151 (17.4)	
Hypertensive nephropathy	61 (6.6)	69 (7.1)	73 (8.4)	
Polycystic kidney disease	37 (4.0)	36 (3.7)	39 (4.5)	
Unknown or miscellaneous	480 (51.6)	508 (52.1)	452 (41.8)	
Smoking history	179 (18.2)	209 (20.3)	238 (25.7)	< 0.001
Hypertension history	819 (83.4)	862 (83.7)	792 (85.5)	0.39
Diabetes mellitus history	125 (12.7)	180 (17.5)	195 (21.1)	< 0.001
Pre-TPL RDW (%)	13.3 (12.7-14.0)	13.6 (12.9-14.5)	13.9 (13.1-14.7)	< 0.001
Laboratory tests at post-TPL 3 months				
Anemia-related tests				
[a]Hemoglobin (g/dL)	12.8 (11.6-13.7)	12.3 (11.2-13.4)	11.6 (10.3-12.9)	< 0.001
[b]Anemia	414 (42.1)	565 (54.9)	650 (70.2)	< 0.001
MCV (fL/red cell)	93.9 (90.4-97.2)	94.4 (91.1-98.1)	96.4 (91.6-101.8)	< 0.001
Iron (μg/dL)	77 (51-106)	76 (44-106)	62 (37-95)	0.10
Ferritin (μg/L)	244 (88-375)	276 (142-470)	473 (200-866)	< 0.001
TIBC (μg/dL)	250 (213-276)	235 (201-269)	223 (179-266)	< 0.001
TSAT (%)	32.0 (18.1-39.1)	29.2 (21.6-44.0)	30.1 (17.8-44.5)	0.96
Iron deficiency (< 20 %)	11 (26.8)	12 (23.5)	33 (31.7)	0.55
Serum creatinine (mg/dL)	1.30 (1.06-1.60)	1.20 (1.00-1.52)	1.27 (1.00-1.60)	0.06
eGFR (mL/min/1.73m^2)	57.4 (43.0-85.5)	57.9 (42.9-84.4)	55.1 (41.2-79.7)	0.05
≥ 60	455 (46.3)	479 (46.6)	396 (42.9)	
30-60	451 (45.9)	466 (45.4)	437 (47.3)	
< 30	77 (7.8)	82 (8.0)	90 (9.8)	
Albumin (g/dL)	4.2 (3.9-4.4)	4.0 (3.8-4.3)	3.9 (3.6-4.2)	< 0.001
Hypoalbuminemia (<3.0 g/dL)	5 (0.5)	7 (0.8)	40 (4.7)	< 0.001
Donor characteristics				
Age (years)	39 (30-48)	40 (30-48)	40 (31-49)	0.52
Sex (male)	402 (41.9)	434 (42.9)	393 (43.8)	0.72

Donor relationship				0.09
Living related	540 (55.3)	533 (52.5)	453 (49.7)	
Living unrelated	220 (22.5)	253 (24.9)	220 (24.1)	
Deceased	216 (22.1)	230 (22.6)	239 (26.2)	
TPL related characteristics				
ABO mismatch	81 (8.4)	65 (6.4)	67 (7.4)	0.26
Positive cross-match	39 (4.0)	31 (3.0)	25 (2.7)	0.26
Acute rejection within 3 months	58 (5.9)	92 (8.9)	96 (10.4)	0.001
Number of HLA mismatch				0.003
Full match	123 (13.1)	96 (9.7)	75 (8.4)	
Mismatch 1-3	483 (51.5)	512 (51.8)	439 (49.4)	
Mismatch 4-6	332 (35.4)	380 (38.5)	375 (42.2)	
Medication use of				
Tacrolimus	523 (53.2)	465 (45.1)	373 (40.3)	< 0.001
Cyclosporine	408 (41.5)	476 (46.2)	429 (46.3)	0.05
Azathioprine	118 (12.0)	157 (15.2)	158 (17.1)	0.007
Induction therapy	553 (56.3)	531 (51.6)	553 (59.7)	0.001

RDW, red cell distribution width, TPL, transplantation, ESRD, end-stage renal disease, MCV, mean corpuscle volume, TIBC, total iron binding capacity, TSAT, transferrin saturation, eGFR, estimated glomerular filtration rate, HLA, human leukocyte antigen.

Categorical variables were presented as n (%), and continuous variables were shown as median scores (interquartile ranges).

[a]The hemoglobin values were measured in the same complete blood cell panel exam which reported the RDW values.

[b]Presence of anemia was defined with hemoglobin level < 12 g/dL for women and < 13g/dL for men.

Supplementary Table 2. Risk of composite graft loss of patients with RDW increment, in subgroups divided by presence of post-transplant anemia.

	Univariable analyses		Multivariable analyses	
	HR (95% CI)	P	[a]Adjusted HR (95% CI)	P
[b]Subgroup w/o anemia (n= 1310)				
Increased RDW (> 14.9%)	1.87 (1.40-2.50)	< 0.001	1.58 (1.16-2.17)	0.003
Higher RDW (1 % increment)	1.17 (1.10-1.25)	< 0.001	1.15 (1.06-1.24)	< 0.001
[b]Subgroup /w anemia (n= 1629)				
Increased RDW (> 14.9%)	1.97 (1.22-3.18)	0.005	1.51 (1.04-2.82)	0.03
Higher RDW (1 % increment)	1.21 (1.08-1.36)	0.001	1.20 (1.05-1.37)	0.007

HR, hazard ratio, CI, confidence interval, RDW, red cell distribution width

Missing data was imputed by multiple imputation by classification and regression trees (CART) method. Hazard ratio and associated confidence of each characteristic was shown. Laboratory values at post-TPL 3 month were used.

[a]Adjusted for baseline characteristics which showed significant differences in patients with increased (> 14.9 %) RDW levels; age (continuous, years), sex, body mass index, smoking history, decreased eGFR (categorical, <30, ≥30), primary glomerulopathy as the cause of ESRD, hemoglobin (continuous, mg/dL), hypoalbuminemia (categorical, serum albumin < 3.0 g/dL), presence of acute rejection (within post-TPL 3 month), donor relationship (deceased or living), number of HLA mismatch (continuous, one antigen increment), whether induction therapy was performed, use of tacrolimus.

[b]Presence of post-TPL anemia was defined by hemoglobin level < 12 g/dL for women and < 13 g/dL for men, and the hemoglobin values were from the same complete blood cell panel exam which reported the RDW levels.

Supplementary Table 3. Risk of composite graft loss of patients with RDW increment, in subgroups divided by usage of RBC transfusion or erythropoietin treatment.

	Univariable analyses		Multivariable analyses	
	HR (95% CI)	P	[a]Adjusted HR (95% CI)	P
Subgroup /w RBC transfusion or erythropoietin treatment (n= 941)				
Increased RDW (> 14.9%)	2.09 (1.47-2.97)	< 0.001	1.59 (1.07-2.37)	0.02
Higher RDW (1 % increment)	1.18 (1.09-1.28)	< 0.001	1.13 (1.02-1.26)	0.02
Subgroup w/o RBC transfusion or erythropoietin treatment (n= 1998)				
Increased RDW (> 14.9%)	1.85 (1.33-2.57)	< 0.001	1.49 (1.04-2.13)	0.03
Higher RDW (1 % increment)	1.19 (1.10-1.29)	< 0.001	1.16 (1.06-1.27)	0.002

HR, hazard ratio, CI, confidence interval, RDW, red cell distribution width

Missing data was imputed by multiple imputation by classification and regression trees (CART) method. Hazard ratio and associated confidence of each characteristic was shown. RDW values at post-TPL 3 month were used, and treatment history with RBC transfusion or erythropoietin within 3 months after TPL was implemented to divide the subgroups.

[a]Adjusted for baseline characteristics which showed significant differences in patients with increased (> 14.9 %) RDW levels; age (continuous, years), sex, body mass index, smoking history, decreased eGFR (categorical, <30, ≥30), primary glomerulopathy as the cause of ESRD, hemoglobin (continuous, mg/dL), hypoalbuminemia (categorical, serum albumin < 3.0 g/dL), presence of acute rejection (within post-TPL 3 month), donor relationship (deceased or living), number of HLA mismatch (continuous, one antigen increment), whether induction therapy was performed, use of tacrolimus.

Supplementary table 4. The association between post-TPL hemoglobin levels and DWGF/DCGF.

	Univariable analyses		Complete case analyses		[a]Models with missing imputation	
	HR (95% CI)	P	[b]Adjusted HR (95% CI)	P	[b]Adjusted HR (95% CI)	P
DWGF						
[c]Hemoglobin increment 1 g/dL						
Values at 3 months after TPL	0.82 (0.74-0.91)	< 0.001	0.92 (0.80-1.06)	0.22	0.93 (0.81-1.05)	0.25
[d]Time-averaged, 3-12 months	0.88 (0.76-1.01)	0.08	0.91 (0.76-1.08)	0.27	0.91 (0.71-1.08)	0.27
DCGF						
[c]Hemoglobin increment 1 g/dL						
Values at 3 months after TPL	0.93 (0.86-0.99)	0.03	0.95 (0.87-1.04)	0.28	0.96 (0.89-1.04)	0.35
[d]Time-averaged, 3-12 months	0.91 (0.83-0.97)	0.007	0.90 (0.82-0.99)	0.03	0.90 (0.82-0.98)	0.02

HR, hazard ratio, CI, confidence interval, DWGF, death-with-graft-function, DCGF, death-censored graft failure, TPL, transplantation

[a]Multiple imputation by CART (classification and regression trees) was performed to impute the missing data.

[b]Adjusted for age (continuous, years), sex, smoking history, eGFR (categorical, <30, 30-60, ≥60), post-transplant RDW (continuous, %), hypoalbuminemia (categorical, serum albumin < 3.0 g/dL), presence of acute rejection (within post-TPL 3 month), baseline diabetes mellitus, hypertension, whether induction therapy was performed, medication use of tacrolimus, donor relationship (deceased)

[c]The hemoglobin values were measured in the same complete blood cell panel exam which reported the RDW values.

[d]Calculated time-averaged values (hemoglobin, eGFR, albumin and RDW) between 3-12 months from operation were used in the analyses. In the analyses using the time-averaged values, the mortality or graft failure cases before 12 months were not included.

Supplementary table 5. Association of RDW change from pre-transplantation to post-transplant 3 months with kidney transplantation prognosis.

	Univariable analyses		[a]Multivariable analyses – model 1.		[b]Multivariable analyses – model 2.	
Effect of 1% increment of RDW change	HR (95% CI)	P	Adjusted HR (95% CI)	P	Adjusted HR (95% CI)	P
On DWGF	1.25 (1.15-1.36)	< 0.001	1.17 (1.06-1.28)	0.002	1.18 (1.08-1.30)	< 0.001
On DCGF	1.16 (1.09-1.24)	< 0.001	1.15 (1.07-1.23)	< 0.001	1.17 (1.09-1.26)	< 0.001

HR, hazard ratio, CI confidence interval, RDW, red cell distribution width, DWGF, death-with-graft-function, DCGF, death-censored graft failure

Those without available pre-TPL RDW levels (59 cases) were not considered in the analyses.

For missing values, multiple imputation by CART (classification and regression tress) method was performed.

[a]Adjusted for age (continuous, years), sex, smoking history, eGFR (categorical, <30, 30-60, ≥60), post-transplant RDW (continuous, %), hypoalbuminemia (categorical, serum albumin < 3.0 g/dL), presence of acute rejection (within post-TPL 3 month), baseline diabetes mellitus, hypertension, whether induction therapy was performed, medication use of tacrolimus, donor relationship (deceased)

[b]Adjusted for same variables as model 1, except for that hemoglobin change from pre-transplant period to 3 months after surgery was adjusted rather than the absolute hemoglobin level at post-transplant 3 months.

Supplementary table 6. Difference of iron, ferritin, TIBC, TSAT levels according to presence of increased RDW at pre- or post-transplantation period.

	Pre/post-TPL RDW ≤ 14.9 % (N=1941)	only pre-TPL RDW > 14.9 % (N=319)	only post-TPL RDW > 14.9 % (N=547)	Pre/post-TPL RDW > 14.9 % (N=132)	P
Post-transplant 3 months					
Iron (μg/dL)	77.5 (50.0-111.0)	53.0 (37.0-79.0)	68.0 (40.5-94.5)	45.0 (36.0-105.0)	0.02
Ferritin (μg/L)	269.0 (158.5-470.4)	216.0 (71.5-393.9)	630 (230.4-1017.0)	388.1 (112.0-805.3)	< 0.001
TIBC (μg/dL)	241.0 (208.0-272.0)	257.0 (201.5-279.0)	217.0 (182.0-261.0)	230.0 (158.0-259.0)	0.005
TSAT (%)	31.4 (21.1-45.6)	28.7 (13.8-31.9)	31.3 (19.2-47.5)	20.1 (15.1-40.1)	0.08
Iron deficiency (< 20%)	20 (22.5)	8 (38.1)	17 (26.6)	11 (50.0)	0.05
Time-averaged values in post-transplant 3-12 months					
Iron (μg/dL)	72.0 (44.2-100.0)	63.0 (49.1-87.2)	73.0 (46.0-104.0)	47.5 (37.0-105.0)	0.64
Ferritin (μg/L)	228.0 (90.5-464.8)	220.5 (87.5-426.9)	547.0 (201.3-957.0)	332.2 (94.2-1050.9)	< 0.001
TIBC (μg/dL)	242.0 (206.0-276.0)	248.5 (197.5-278.0)	217.0 (179.6-254.0)	231.5 (174.0-266.5)	0.001
TSAT (%)	27.5 (18.5-40.2)	29.6 (19.7-36.3)	32.9 (21.1-54.1)	21.9 (15.1-43.0)	0.20
Iron deficiency (< 20%)	52 (29.9)	10 (25.6)	24 (23.5)	11 (36.7)	0.47

RDW, red cell distribution width, TPL, transplantation, TIBC, total iron binding capacity, TSAT, transferrin saturation

Categorical variables were presented as n (%), and continuous variables were shown as median scores (interquartile ranges).

Supplementary table 7. Documented post-TPL complications in the study population.

	Pre/post-TPL RDW ≤ 14.9 % (N=1941)	only pre-TPL RDW > 14.9 % (N=319)	only post-TPL RDW > 14.9 % (N=547)	Pre/post-TPL RDW > 14.9 % (N=132)	P
N of patients with infection					
Urinary tract infection	151 (7.8)	20 (6.3)	73 (13.4)	11 (8.3)	< 0.001
Pneumonia	74 (3.8)	3 (0.9)	45 (8.2)	11 (8.3)	< 0.001
Cytomegalovirus	55 (2.8)	8 (2.5)	52 (9.5)	5 (3.8)	< 0.001
BK virus or parvovirus	31 (1.6)	8 (2.6)	16 (2.9)	1 (0.8)	0.13
N of patients with malignancy	42 (2.2)	4 (1.3)	17 (3.1)	4 (3.0)	0.30

RDW, red cell distribution width, TPL, transplantation

Categorical variables were presented as n (%).

Supplementary Figure 1. Post-transplant prognoses according to tertile RDW ranges.

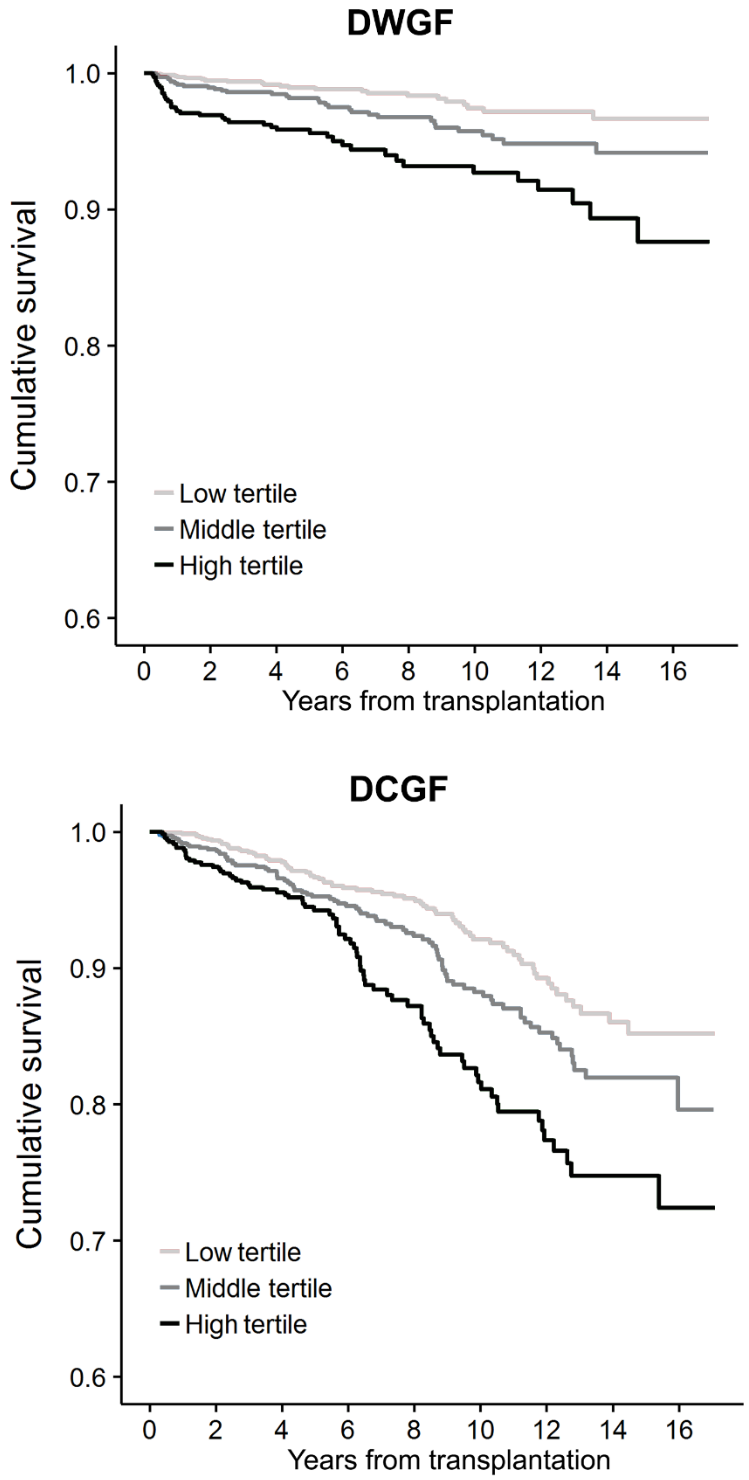

Cumulative survival curve of the study population, y-axis indicated the cumulative survival and x-axis indicated the years from transplantation. The upper graph shows the DWGF, and the lower graph shows the DCGF. Black lines, dark grey lines, and light grey lines indicate the survival curve of patients with high tertile (≥14.6), middle tertile(<14.6 and ≥13.4), and low tertile (<13.4%) RDW ranges, respectively. RDW, red cell distribution width; DWGF, death-with-graft-function, DCGF, death-censored-graft-failure.
